# The Mitochondrial Aminoacyl tRNA Synthetases: Genes and Syndromes

**DOI:** 10.1155/2014/787956

**Published:** 2014-02-04

**Authors:** Daria Diodato, Daniele Ghezzi, Valeria Tiranti

**Affiliations:** Unit of Molecular Neurogenetics, Pierfranco and Luisa Mariani Center for the Study of Mitochondrial Disorders in Children, IRCCS Foundation Neurological Institute “C. Besta”, Via Temolo 4, 20126 Milan, Italy

## Abstract

Mitochondrial respiratory chain (RC) disorders are a group of genetically and clinically heterogeneous diseases. This is because protein components of the RC are encoded by both mitochondrial and nuclear genomes and are essential in all cells. In addition, the biogenesis and maintenance of mitochondria, including mitochondrial DNA (mtDNA) replication, transcription, and translation, require nuclear-encoded genes. In the past decade, a growing number of syndromes associated with dysfunction of mtDNA translation have been reported. This paper reviews the current knowledge of mutations affecting mitochondrial aminoacyl tRNAs synthetases and their role in the pathogenic mechanisms underlying the different clinical presentations.

## 1. Introduction

Mitochondria are double-membrane cytoplasmic organelles essential for energy supply to the cell. Adenosine-5′-triphosphate (ATP), the molecular unit of currency for intracellular energy transfer, is produced by the last of five multisubunit complexes embedded in the inner mitochondrial membrane, which form the respiratory chain (RC) responsible for oxidative phosphorylation (OXPHOS).

Mitochondria have their own DNA (mtDNA) that is a circular, double-stranded molecule; in humans, it is 16.569 base pairs long and contains genes encoding 13 protein subunits of RC complexes I, III, IV, and V as well as transfer (t) and ribosomal (r) RNA encoding genes for mtDNA-specific translation. However, hundreds of additional gene products, providing the components necessary for mtDNA replication and expression, many RC complex subunits, and the complex protein network needed for RC formation, activity, and turnover, are nuclear-encoded.

The term mitochondrial disorder refers to diseases that are caused by OXPHOS dysfunction and comprises a clinically and genetically heterogeneous group of syndromes that all together are amongst the most common inherited human diseases, with a prevalence of 1 : 5000.

Deficiencies of single RC complex are generally caused by mutations in genes encoding structural subunits or proteins involved in the assembly of a specific OXPHOS enzyme complex. On the contrary, combined OXPHOS defects are often associated with impairment of processes such as replication, transcription, or translation of mtDNA, which can be due to mutations in either mtDNA-encoded RNAs (tRNAs and rRNAs) or nuclear DNA-encoded proteins [1, 2]. Hundreds of RNA and protein factors have been maintained through evolution of eukaryotes to carry out the synthesis of a few, but essential, mtDNA-encoded proteins, carried out in situ by organelle-specific translation machinery [3].

Given the multitude of proteins required for proper mitochondrial protein synthesis, it is not surprising that mitochondrial disorders due to impairment of this essential process are genetically heterogeneous and that, in many patients, the molecular genetic defect remains unknown.

This review will focus on a group of enzymes with a key role in mitochondrial protein synthesis, the aminoacyl tRNA synthetases (mt-aaRSs), mutations of which are responsible for an increasing number of OXPHOS deficiencies and diseases (Table 1).

## 2. Mitochondrial Protein Synthesis

Protein synthesis is a complex process that in mitochondria supplies the mtDNA-encoded subunits of RC complexes through an organellar-specific translational apparatus distinct from the cytosolic counterpart. In fact, there are some distinguishing features for the mitochondrial translation, including the mitochondrial genetic code, which differs from the universal one (i.e., AUA codes for methionine and UGA, instead of being a stop codon, codes for tryptophan), and the structure of mitochondrial mRNAs, which have no or few 5′ untranslated nucleotides, is uncapped and contains a poly (A) immediately after the stop codon.

The human mitoribosome is made up of 2 ribosomal RNAs (rRNA) and 81 mitochondrial ribosomal proteins (MRPs) and comprises two subunits, the small (SSU or 28S) and the large (LSU or 39S) [3]. Mammalian mitoribosome differs markedly from bacteria, cytosolic, and also from other mitochondrial ribosomes, due to the peculiar recruitment of numerous extraproteins. Most of the protein components involved in mitochondrial translation such as a multitude of initiation, elongation and termination factors, subunits and assembly protein of mitoribosome, tRNA-modifying enzymes, and the mt-aaRSs are encoded by nuclear genes, whereas 2 mitochondrial ribosomal and 22 transfer RNAs (rRNAs and tRNAs) are encoded by mtDNA [4]. Mitochondrial mRNA translation has been hypothesized to take place in a complex bound to the inner membrane through electrostatic force and maybe protein interaction [5]. The basic model of mitochondrial protein synthesis is derived from studies in bacteria and can be divided into three phases: initiation, elongation, and termination [3, 6].

Due to the unusual characteristics of mitochondrial mRNA, it is not fully clear how the protein synthesis starts. It is thought that mitochondrial initiation factor mtIF3 (the ortholog of prokaryotic IF3) induces the dissociation of the mitoribosome into its two components, the small (SSU or 28S) and the large (LSU or 39S) subunits. This facilitates the binding of the mRNA to the SSU subunit directing the ribosome to the start codon. Mammalian mitochondria use a single tRNA^Met^ for both the initiation and elongation phases; after aminoacylation, the tRNA^Met^ needs to be formylated by methionyl-tRNA transformylase (forming fMet-tRNA^Met^) to initiate mitochondrial translation. A second mitochondrial initiation factor, mtIF2, is required for binding of the fMet-tRNA^Met^ to the SSU. Recombining of the mitoribosome subunits and GTP hydrolysis promote the release of mtIF3 and mtIF2 and the completion of the initiation phase.

In the elongation phase, a crucial role is played by the elongations factors mtEFTu, mtEFTs, and mtEFG (1 and 2). mtEFTu forms a complex with GTP and a tRNA charged with its corresponding amino acid, protecting the latter from hydrolysis and facilitating the codon-anticodon recognition at the acceptor site A of the SSU. This step requires GTP hydrolysis and leads to the release of mtEFTu. The aminoacyl-tRNA moves into the peptidyl (P) site of the mitoribosome, where the amino acid is added to the growing peptide. mtETFs recycles the released mtEFTu-GDP, reforming an active mtEFTu-GTP. mtEFG1 catalyzes the translocation of tRNAs from P to exit (E) site of the mitoribosome, while mRNA is advanced by one codon. This last step has been derived from the prokaryotic model; the E site has been suggested to be very weak or even absent in the mitoribosome. Finally, the tRNA leaves the mitoribosome and a new elongation cycle can start. The functional role of mtEFG2 in mitochondrial translation is not completely understood.

The termination phase initiates with the recognition of a stop codon by the mitochondrial release factor (mtRF1 or mtRF1a). This causes the detachment of the polypeptide linked to the last tRNA present in the P site. After release of the newly synthesized protein, mitochondrial ribosome recycling factors (mtRRF and possibly mtEFG2) enable the mitoribosomal subunits, tRNA and mRNA, to dissociate from each other, allowing the reuse of them for a new round of protein synthesis [7, 8].

Mitochondrial disorders with different clinical presentation have been associated with defects in several enzymes involved in mitochondrial translation including tRNA-modifying enzyme like TRMU (tRNA 5-methylaminomethyl-2-thiouridylate methyltransferase, OMIM#613070) [9] and MTO1 (Mitochondrial Translation Optimization 1, OMIM#614702) [10] or elongation factors like GFM1/EFG1 (OMIM#609060), TSFM/EFTs (OMIM#610505), and TUFM/EFTu (OMIM#610678) [11, 12].

## 3. Aminoacyl-tRNA Synthetases 

Aminoacyl-tRNA synthetases (aaRSs) are key enzymes in the translation of the genetic information. In fact, they catalyze the specific attachment of each of the 20 amino acids (aa) to a cognate tRNA, through a two-step reaction, where they first activate the amino acid with ATP, forming an intermediate aminoacyl-adenylate, and then transfer the aminoacyl group to the 3′-end of its own tRNA [3, 13].

With the exception of GARS and KARS, mitochondrial and cytoplasmic aaRSs are encoded by distinct nuclear genes. The gene for mitochondrial glutaminyl-tRNA synthetase has not been found yet, possibly because mitochondrial glutamyl-tRNA synthetase (EARS2) efficiently misaminoacylates mt tRNA^Gln^ to form glutamate charged-tRNA^Gln^ [14]. Hence, mt-aaRSs are 19 instead of 20, including the 2 with double localization.

Mt-aaRSs are encoded by nuclear genes (*aaRS2*), imported into mitochondria, and their interaction with cognate tRNAs seems to be essential for their amount and stability [15]. Mt-tRNA genes are located in three transcription units: the short H-strand unit (with the rRNA region and two tRNA genes) is transcribed twice as fast as the L-strand that contains eight tRNA genes and more frequently then the entire H-strand unit that produces 14 tRNAs. King and Attardi showed that steady-state levels of different tRNAs remain uniform suggesting a mechanism of posttranscriptional regulation of redundant tRNAs [16].

aaRSs structure typically presents a catalytic domain and an anticodon binding domain; some of them have also an editing domain to deacylate mischarged amino acids, preventing insertion of incorrect amino acids during protein synthesis [17]. These enzymes can be classified into two distinct classes according to the structure of the catalytic site: class I enzymes bear the classical Rossmann fold (defined as an adenylic-nucleotides recognition site) that displays five parallel *β*-strands connected via *α*-helices and the two signature motifs; class II enzymes display an alternate folding, mainly constituted by a sheet of six antiparallel *β*-strands and three motifs of less-conserved sequences [18].

During evolution, aaRSs have acquired additional domains and insertions thus expanding their range of function. Cytosolic aaRSs have been discovered to play many other roles with either extracellular (cytokines or angiogenesis regulators) or cytoplasmic activities being involved in apoptosis, synthesis of rRNA, or tRNA export to the cytosol. On the other hand, also mt-aaRSs have been investigated for having additional functions; for instance, it has been demonstrated that the yeast homologue of human mitochondrial lysyl-tRNA synthetase is a dual function protein, involved in both aminoacylation of mitochondrial tRNA^Lys^ and import of cytosolic tRNA^Lys^ into mitochondria[19].

## 4. Aminoacyl-tRNA Synthetases and Mitochondrial Disorders

An increasing fraction of mitochondrial protein synthesis deficiencies are caused by mutations in one of the *aaRS2* genes and have been associated with diverse clinical presentations, usually with an early onset and transmitted as autosomal recessive traits. Such a broad clinical spectrum can be partially explained by the multiple and still unknown functions of mt-aaRSs. However, there is a strict genotype-phenotype correlation for most of these genes, albeit the reason for specific and different cellular or tissue damage, being all aaRSs ubiquitous enzymes working in the same pathway is not clear. Because of the fundamental function of these enzymes, it has been suggested that mt-aaRSs loss of function mutations is not compatible with extrauterine life and that a residual enzymatic activity can result in different tissue alterations after development [20].

We present an overview of the main clinical presentations associated with mutations in *aaRS2s*, which have been identified in the last 10 years, initially by linkage mapping/homozygosity mapping and candidate gene analysis and, more recently, by next-generation whole exome sequencing (WES).

### 4.1. *DARS2*

*DARS2,* encoding the mitochondrial aspartyl-tRNA synthetase (mt-AspRS), was the first *aaRS2* gene reported to cause a human disease, characterized by a peculiar leukoencephalopathy named LBSL (leukoencephalopathy with brain stem and spinal cord involvement, OMIM #611105) [21] associated with cerebellar ataxia, spasticity, and variable degree of cognitive impairment. To date, more than 30 families have been described with mutations in *DARS2* (Table 1). In mutant patients, high lactate has been observed only in the affected white matter. White matter changes, involving brain stem and spinal cord tracts, are very peculiar. Almost all patients with LBSL are compound heterozygotes, sharing a complex rearrangement in one allele that involves a T-C stretch upstream from exon 3 (228-20/-21delTTinsC) and a second variable, usually missense, mutation. The splice site mutation in intron 2 is a hypomorphic mutation that partially interferes with the splicing of exon 3, leading to frameshift and premature truncation (Arg76SerfsX5) of only a fraction of *DARS2* transcripts, maintaining some mt-AspRS residual activity. Van Berge et al. tried to explain the brain tissue specificity showing differences in mRNA splicing process between neurons and other cells [22]. Some compound heterozygous patients have been reported to show atypical presentations including specific MRI pattern associated with no clinical symptoms [23] or no lactate elevation [24]. Axonal neuropathy was an important and frequent feature of LBSL [25]. Homozygous *DARS2* mutations have been shown to cause a more severe phenotype [26, 27] or exercise-induced paroxysmal gait ataxia and areflexia [28].

Surprisingly, blue-native PAGE as well as spectrophotometric measurements often revealed normal OXPHOS enzyme activities in muscle and cultured cells.

### 4.2. *RARS2*

Mutations in *RARS2* gene have been described so far in more than 10 families (Table 1). The first patients described with mutations in *RARS2, *the gene encoding for the mitochondrial arginyl-tRNA synthetase (mt-ArgRS), presented progressive atrophy in cerebellum, pons (pontocerebellar hypoplasia type 6, PCH6; OMIM#611523), with cerebral cortex and white matter involvement, causing a severe infantile clinical picture characterized by lethargy, seizures with apneic spells, hypotonia, and premature death. These consanguineous Sephardic Jewish patients harboured a homozygous splice site variant (IVS2 +5A>G) [29] and showed multiple RC defects in muscle and fibroblasts. However, at least three *RARS2* mutant patients, carrying missense mutations, have been reported without any RC defect in muscle [30, 31]. Later, it has been demonstrated that the subtype 1 of pontocerebellar hypoplasia (OMIM#607596), characterized by a neuropathological profile with loss of spinal anterior horn cells, diffuse gliosis, flat cerebellar folia, loss of Purkinje cells, and pontocerebellar hypoplasia, can be also caused by mutation in *RARS2 *[32]. Recently, compound heterozygous *RARS2* mutations have been described in patients with neonatal onset epileptic encephalopathy and typical PCH6 MRI findings. These mutations have been shown to cause reduced RARS2 amount and activity, associated with impaired tRNA^Arg^ stability [31].

### 4.3. *YARS2*

*YARS2* encodes for the mitochondrial tyrosyl-tRNA synthetase (mt-TyrRS). Only two missense mutations in this gene have been reported till now, associated with myopathy, lactic acidosis, and sideroblastic anemia (MLASA, OMIM#613561). These mutations, present in homozygous state in different patients, affect the enzyme catalytic domain causing generalized mitochondrial translation defect in myoblasts [33]. Recently, a more severe phenotype has been described associated with the known homozygous mutation c.156C>G; p.Phe52Leu [34]. Complexes I, III, and IV were defective in skeletal muscle but not in fibroblasts. Gel filtration experiments showed that mt-TyrRS is part of high-molecular-weight complexes (*∼*250 kDa and 1 MDa), suggesting that, like some of the cytoplasmic aaRSs, mt-aaRSs could also occur in high-molecular-weight complexes [35].

### 4.4. *SARS2*

Belostotsky et al. [36] described three probably related Palestinian infants with a homozygous mutation c.1169A>G (p.Asp390Gly) in *SARS2*, the gene encoding the mitochondrial seryl-tRNA synthetase (mt-SerRS). They were affected by a multisystem disorder, characterized by prematurity, progressive renal failure leading to electrolyte imbalances, metabolic alkalosis, and pulmonary hypertension, called HUPRA syndrome (Hyperuricemia, pulmonary hypertension, renal failure, and alkalosis, OMIM#613845), and died within the first 14 months of life. Global developmental delay was an additional feature.

Mt-tRNA^Ser^ catalyzes the ligation of serine to two mitochondrial tRNA isoacceptors: tRNA^Ser(AGY)^ and tRNA^Ser(UCN)^. The Asp390Gly mutation impacts the acylation of tRNA^Ser(AGY)^ but probably not that of tRNA^Ser(UCN)^ with severe decrease in the expression of the nonacylated transcript and the complete absence of the acylated tRNA^Ser (AGY)^.

Recently, a novel SARS2 homozygous mutation c.1205G>A (p.Arg402His) has been identified in a girl and her brother with HUPRA syndrome [37].

### 4.5. *AARS2*

*AARS2* encodes the mitochondrial alanyl-tRNA synthetase (mt-AlaRS). All alanyl-tRNA synthetase orthologs have an aa binding pocket equally accessible by alanine, serine, and glycine; hence, this enzyme contains an editing domain, in addition to the catalytic one, essential to deacylate the mischarged tRNAs. In 2011, Götz et al. [38] described *AARS2* mutations causing hypertrophic cardiomyopathy and lactic acidosis in infants from two different families, one being homozygous for the p.Arg592Trp mutation and the other being compound heterozygous for the p.Arg592Trp and the missense p.Leu155Arg. All patients died at few months of age; they showed a drastic deficiency of RC complexes I, III, and IV in heart tissue, with less severe defects in brain and skeletal muscle (OMIM#612035). Patients' fibroblasts or myoblasts did not show any OXPHOS defect. p.Arg592Trp localizes in the editing domain, thus probably affecting the recognition of mischarged tRNAs, while the other missense mutation is located in the aminoacylation domain.

*AARS2* mutations can be also associated with a different clinical presentation, characterized by cerebellar ataxia and psychotic features with leukoencephalopathy [39].

### 4.6. *MARS2*

Bayat et al. [40] described mutations in *MARS2* encoding the mitochondrial methionyl-tRNA synthetase (mt-MetRS), which cause autosomal recessive spastic ataxia with leukoencephalopathy (ARSAL or spastic ataxia 3, OMIM#611390) in humans and neurodegeneration in flies. Patients' brain MRI showed cerebellar atrophy and white matter alterations sometimes associated with thin corpus callosum; disease onset was very variable. In all ARSAL subjects, complex gene rearrangements were identified in *MARS2*, including homozygous duplication or compound heterozygous duplication-deletion (in this last case, disease onset was anticipated). Despite increased levels of aberrant *MARS2* transcripts, protein levels were lower in all patients. Furthermore, patients' fibroblasts displayed a reduced complex I activity as in mutant flies and immortalized lymphoblast lines showed an impaired translation [40].

### 4.7. *HARS2*

Mutations in *HARS2*, encoding the mitochondrial histidyl-tRNA synthetase (mt-HisRS), have been associated with Perrault syndrome in a family with five members affected (OMIM#614926). Perrault syndrome is a clinical entity characterized by ovarian dysgenesis and sensorineural hearing loss. This recessive syndrome is genetically heterogeneous, being caused by mutations in different genes (*HSD17B4*, *CLPP,* and *LARS2*, besides *HARS2*), and is associated with premature ovarian failure in females and progressive hearing loss in both males and females. The *HARS2* mutant patients were compound heterozygous for two missense mutations, p.Val368Leu and p.Leu200Val, the latter creating an alternative splice site that causes an in-frame deletion of 12 codons. Both missense mutations caused significantly decreased enzyme activity compared to wild type. The enzymatic defect and the oxidative phenotype in the yeast model were more severe for the p.Val368Leu mutation [41].

### 4.8. *LARS2*

More recently homozygous mutations in *LARS2*, encoding the mitochondrial leucyl-tRNA synthetase (mt-LeuRS), have been found in patients with Perrault syndrome (OMIM#615300). Three affected subjects from a consanguineous Palestinian family carried a homozygous p.Thr522Asn substitution of a highly conserved residue in the catalytic domain, whereas compound heterozygous mutations were found in a Slovenian woman [42]: a c.1866C>T transition resulting in a p.Thr629Met substitution at a residue conserved in mammals, and a 1-bp deletion (c.1077delT) predicted to yield a 373-amino-acid truncated protein with 14 novel amino acids at the C terminus (p.Ile360PhefsTer15). Analysis in yeast models suggested reduced activity relative to wild type for the identified mutations [42].

### 4.9. *FARS2*

Mutations in the mitochondrial phenylalanine-tRNA synthetase, FARS2 (mt-PheRS), have been identified by WES in two couples of siblings, characterized by fatal epileptic encephalopathy, liver disease, and lactic acidosis. Neuropathological findings together with liver disease filled the criteria for Alpers-Huttenlocher syndrome (OMIM#614946). All *FARS2* mutant patients died within the first two years of life. The Saudi siblings were homozygous for a homozygous 431A-G transition, resulting in a Tyr144Cys (Y144C) substitution at a highly conserved residue in the catalytic domain [43]. The Finnish siblings were compound heterozygous for two missense variants, c.986T>C (p.Ile329Thr) and c.1172A>T (p.Asp391Val), affecting residues in the ATP-binding site in the aminoacylation domain, or in the anticodon stem-binding domain, respectively [44]. Blue-native gel electrophoresis showed a defective complex IV in both brain and muscle, whereas complex I was decreased only in brain. Patients' fibroblast displayed neither biochemical defects nor impaired mitochondrial translation [44].

Recently, a partial genomic deletion and a p.Asp325Tyr missense mutation in *FARS2* have been described in a patient with early-onset epilepsy and isolated complex IV deficiency in muscle. The biochemical defect was found in myoblasts but not in fibroblasts and was associated with decreased steady-state levels of COXI and COXII proteins and reduced steady-state levels of the mt-tRNA^Phe^ transcript [45].

### 4.10. *EARS2*

*EARS2* encodes for the mitochondrial glutamyl-tRNA synthetase (mtGluRS). Mutations in this gene have been identified by WES in a single Italian patient and then found in 11 additional patients with similar MRI alterations, described as LTBL (leukoencephalopathy with thalamus and brain stem involvement and high lactate, OMIM#614924) [46]. Only one additional patient with *EARS2 *mutations has been described to date [47]. *EARS2* mutant patients presented a peculiar biphasic clinical course: an infantile disease onset with rapid progression followed by stabilization, and in some cases improvement, and partial recovery of lost skills. MRI displayed abnormal thalami, midbrain, pons, and medulla oblongata and alterations of cerebral and cerebellar white matter. High blood lactate and OXPHOS defects in muscle and fibroblasts were present. All patients were compound heterozygous for a severe mutation and a milder one, suggesting that there is a threshold for mtGluRS activity to cause the disease. The only patient reported with a homozygous severe mutation (p.Lys65Glu) presented a dramatic phenotype with brain and liver involvement and a fatal outcome at age 3 months [47].

### 4.11. *VARS2* and *TARS2*

Recently, mutations in other two mt-aaRSs have been reported as causative of severe recessive infantile disorders with OXPHOS deficiency. A homozygous missense mutation (p.Thr367Ile) in *VARS2*, the gene encoding the mitochondrial valyl-tRNA synthetase (mt-ValRS), has been found in a child with psychomotor delay, seizures, and facial dysmorphisms. Two siblings with phenotype characterized by hypotonia, psychomotor retardation, and premature death were found to be compound heterozygous for a splice site (c.695 +3A>G) and a missense (p.Pro282Leu) mutation in the mitochondrial threonyl-tRNA synthetase gene *TARS2* [39].

### 4.12. *GARS* and *KARS*

In addition to genes coding for mitochondrial-only or cytosolic-only aaRSs, two genes, *GARS* and *KARS,* encode enzymes, glycyl-tRNA synthetase (GlyRS) and lysil-tRNA synthetase (LysRS), which catalyze the reaction in both mitochondria and cytosol.

Dominant *GARS* mutations have been found in patients with Charcot-Marie-Tooth disease type 2D (CMT2D; OMIM#601472) and with distal hereditary motor neuronopathy type VA (HMN5A; OMIM#600794) [48, 49]. Experimental evidence demonstrated a key role for glycine-tRNA synthetase in maintaining peripheral axons [50]. More recently a toxic role of mutant GARS on peripheral nerves has been shown [51]. The dominant transmission can be ascribed to either loss of function (haploinsufficiency) or a dominant-negative loss-of-function effect, but also aberrant pathogenic function of the mutant protein has been hypothesized [52]. Remarkably, most of the known mutations in cytoplasmic ARSs are associated with a CMT phenotype or related neuropathies. Mutations in *YARS* have been described in patients with Dominant Intermediate (DI) CMT type C [53], whereas *AARS *mutations have been found in patients with a CMT2 phenotype [54] and CMT2N [55]; *HARS *mutations have been also associated with axonal peripheral neuropathy [56].

Mutations in *KARS* have been initially described in a patient with an intermediate form of autosomal recessive Charcot-Marie-Tooth disease (CMTRIB; OMIM#613641) [57], developmental delay, self-abusive behavior, dysmorphic features, and vestibular schwannoma. The p.Tyr173SerfsX7 variant represents a loss-of-function allele, and p.Leu133His represents a severely hypomorphic allele in yeast growth and aminoacylation assays, respectively. The presence of compound heterozygosity for a null and severely hypomorphic *KARS* allele may explain the more severe or complex neuropathy phenotype than those found associated with other heterozygous dominant-negative *aaRS* mutation. Very recently, *KARS* homozygous missense mutations have been identified by WES in affected subjects from three consanguineous Pakistani families, with nonsyndromic-hearing-impairment phenotype [58]. No auditory neuropathy and CMT were reported. It has been hypothesized that *KARS* variants affect aminoacylation by interfering with binding activity to tRNA and with formation of the active LysRS tetramer. Because of the proven localization of LysRS within the cochlea, the authors suggested that perturbations in aminoacylation might affect many of the cellular processes of the different specialized cells of the cochlea and therefore result in hearing impairment.

As *GARS* and *KARS* encode for both the mitochondrial and cytoplasmic aaRSs, the evaluation of mitochondrial involvement in these phenotypes is complicated and has not been studied in detail.

## 5. Functional Studies

Given the function of the mt-aaRSs, pathogenic mutations are expected to impair binding between tRNAs and their cognate amino acids, resulting in reduction of charged tRNA, albeit other defects can be envisaged, such as defective mitochondrial targeting. However, analysis of amino acylation is a tricky process that requires a large amount of starting material, and it is rarely performed. Usually the target of the studies is a downstream effect of mt-aaRSs dysfunction, such as altered mitochondrial protein synthesis or OXPHOS deficiency.

The lack of clear biochemical phenotypes (OXPHOS or mitochondrial protein synthesis defects) in skin fibroblasts and myoblasts from most of mutant *aaRS2* patients prevented the use of cultured cells for functional studies. In few *RARS2* patients, fibroblasts showed OXPHOS defects, with RC enzymes variably affected; *YARS2* patients presented deficient mitochondrial protein synthesis in myotubes but not in cultured fibroblasts. Microscale oxygraphy (by Seahorse instrument) could be a more sensible approach to reveal biochemical defects in cell lines: in *EARS2* mutant fibroblasts, microoxygraphic analysis revealed reduction of maximal respiratory rate and reduced oxygen consumption rate to extracellular acidification rate ratio, reflecting increased production of lactate [46]. Otherwise, induced pluripotent stem cells derived from patient cell lines, differentiated into various cell types, may offer advantages in future studies.

The high degree of conservation of several mt-aaRSs during evolution makes feasible the use of user-friendly bacterial and yeast model to evaluate the effects of identified variants on protein function. In some studies, the recombinant mt-aaRSs proteins (i.e., mt-PheRS and mt-AspRS) were expressed and purified in *Escherichia coli*, allowing the comparison of enzyme parameters such as catalytic activity, ATP binding, and protein stability between mutant and wt species. In other works, (i.e., *RARS2, HARS2, LARS2, *and* KARS*) mutations were studied on facultative aerobic yeast *Saccharomyces cerevisiae*. The yeast orthologous gene can be inactivated by homologous recombination and the resulting cells then transformed with a centromeric plasmid containing either the wild type or a mutant allele. Usually, all strains are able to grow in a medium containing a fermentable sugar, but strains with mutations affecting the OXPHOS system show decreased or abolished respiration and display a reduced growth on nonfermentable medium [31]. A restriction in the use of this approach is the high number of yeast *aaRS* genes coding enzymes with a double localization, both mitochondrial and cytosolic [59]. This limitation can be bypassed with a more complex strategy, based on the inactivation of the endogenous bifunctional enzyme and the reexpression of modified plasmids coding specifically either the cytosolic or the mitochondrial enzyme.

At the moment, no mouse model for mt-aaRSs has been fully characterized and published. Only two mutagenesis-induced mouse models for GARS have been described: a dominant model caused by an in-frame indel mutation at pro278 [52] and a second model with a point mutation (C201R) that leads to a nonconservative substitution [60]. The first model presented sensorimotor polyneuropathy with overt neuromuscular dysfunction and shortened life spans. Heterozygous mutant mice showed neurodegenerative changes at the neuromuscular junctions, whereas homozygous mutant mice were embryonic lethal. The dominant transmission was probably due to a gain of function [52]. The second model was less severe but displayed locomotor and sensory deficits, with myelination defects not detected in the former model, representing a valuable resource to study the mechanism of axonopathy resulting from GARS mutations [60].

The group of Aleksandra Trifunovic is currently dealing with *DARS2* mouse models. They recently reported that constitutive Knock-Out (KO) mice had an early developmental arrest with embryonic lethality, whereas conditional cardiac and skeletal muscle KO mice showed dramatically shortened life span and a gradual increase in heart/body weight ratio, with strong reduction of all assembled respiratory chain complexes, except complex II, in both heart and skeletal muscle [61]. Two probably more interesting mouse models are undergoing study, based on different brain-specific *DARS2* depletion in either adult forebrain neurons or in myelin-producing oligodendrocytes.

The production of conditional tissue-specific mouse models for different mt-*aaRSs *could provide an insight into the pathological mechanisms and the selective tissue involvement caused by mutations in these ubiquitously expressed enzymes.

## 6. Discussion

It is hard to explain why genetic defects in mt-aaRSs, which are ubiquitous enzymes necessary for mitochondrial translation, can cause so many different phenotypes usually affecting specific tissues or organs. Different hypotheses have been proposed to explain this variability, but none seems to cover all aspects of this phenomenon, that is probably the result of several different mechanisms altered in mt-aaRSs mutant patients.

The first patients with splice-site mutation in either *DARS2* or *RARS2* presented with encephalopathy. Differences in mRNA splicing machinery, in abundance of splicing factors, and splicing efficiency between neuronal and other cell types have been hypothesized to explain this tissue specificity. However, other patients harbouring *DARS2* or *RARS2* missense mutations have been reported later weakening the general value of this hypothesis. In addition to *DARS2* and *RARS2* mutations, also the tissue specificity present in *HARS2* mutant patients may be influenced by splicing efficiency in different tissues/organs, in the latter case ovaries and ears. However, only one family has been described till now and the identification of other patients with *HARS2* mutations is required to confirm this observation.

Other types of mutations and their location inside the protein could have an influence on the resulting phenotype. For instance, the *AARS2* p.Arg592Trp mutation is located at the surface of the editing domain, which functions in deacylating the mischarged tRNAs, and is predicted to affect the recognition of mischarged tRNAs [38]. The pathogenic mechanism of this mutation may be linked to mitochondrial mistranslation and hence be different from other mt-aaRS defects. Why mistranslation would lead specifically to cardiomyopathy, while translation deficiencies caused by other mt-aaRSs mutations are usually not associated with heart damage remains an open question. Noteworthy, mutations affecting MTO1, an enzyme responsible for tRNA modifications that increases the accuracy and efficiency of mtDNA translation, have been found in patients with hypertrophic cardiomyopathy [62].

Alternatively, the bigger susceptibility of some cell types, in particular neurons, for aminoacyl-tRNA synthetase dysfunction could be linked to the variable expression of mitochondrial tRNAs in different cells, to the amount of cognate amino acid in a particular tissue [38], or to different threshold required for optimal enzymatic activity of each mt-aaRS. The brain expresses higher levels of mitochondrial-encoded tRNAs as compared to other examined tissue, including liver, vulva, testis and ovary, thymus, lymph node, and spleen [63]. Moreover, variations in the relative expression of tRNA isoacceptors among tissues have been observed: thus the tRNA levels could influence the usage of different codons among different tissues [63].

For *AARS2* mutations, the tissue-specific manifestation of the cardiomyopathy could be explained by variable amino acid concentrations in different tissues; in fact, increased levels of alanine have been found in affected tissue (heart and muscle), but not in the unaffected liver. This observation could be due to a compensatory feedback mechanism occurring upon deficient tRNA^Ala^ aminoacylation or be an unspecific response to RC defect reflecting the alanine secreted from RC-deficient skeletal muscle for gluconeogenesis [38].

For some peculiar phenotypes, the pathological mechanisms seem to be specific and unique. In addition to *YARS2* mutations, MLASA can be caused also by mutation in *PUS1*, encoding pseudouridylate synthase 1. This enzyme is probably responsible for pseudouridylation of tRNA^Tyr^. This observation suggests that tRNA^Tyr^ and not a general mitochondrial translation defect is specifically responsible for the combined myopathy and anemia in *YARS2* mutant patients.

*GARS* and *KARS,* encoding enzymes acting both in mitochondria and cytosol, require ad hoc comments and considerations. Few pathological pictures, mainly CMT, have been described caused by defective cytoplasmic aaRSs; hence, the neuropathy phenotypes associated with *GARS* or *KARS* mutations have been ascribed mainly to impairment of the cytoplasmic enzyme. However, the possible contribution of faulty mitochondrial functionality in the development of the phenotype has not been fully analyzed. It should be noted that axonal CMT is frequently caused by mutations in a mitochondrial protein, MFN2, involved in mitochondrial fusion; however, it is not known if and how GlyRS or LysRS could be linked directly to mitochondrial dynamics or how a mitochondrial synthesis deficiency could lead to axonal dysfunction.

While mutations in *GARS* or other cytosolic *aaRSs* are inherited as dominant trait, *KARS* mutation are recessive and the presence of two mutant alleles has been suggested as responsible for the most severe phenotype in these patients, including acoustic neuroma and dysmorphisms besides CMT [57]. More recently, recessive *KARS* mutations have been reported in patients with nonsyndromic hearing impairment without neuropathy, expanding the spectrum of phenotypes associated with *KARS* mutation [58]. Unfortunately, the selective impairment of mitochondrial protein synthesis has not been investigated in these patients. Interestingly, hearing problems are present also in patients with PCH6 (due to *RARS2* mutations) or Perrault syndrome (caused by *LARS2* or *HARS2* mutations). In addition, a number of different mutations in several genes of the mitochondrial genome, mainly *MTRNR1* encoding the mitochondrial ribosomal RNA 12S, and many tRNA-encoding genes, are responsible for hearing impairment or deafness, in some cases associated with a variety of additional clinical features.

Mutations in *DARS, *encoding the cytosolic AspRS, have been recently found in a cohort of patients with a leukoencephalopathy characterized by hypomyelination with brain stem and spinal cord involvement and legs spasticity (HBSL, OMIM#615281) [64]. HBSL resembles LBSL, which is caused by mutations in *DARS2*, suggesting that these two diseases might share a common underlying molecular pathology. Many aaRSs have additional functions beyond translation [19, 65] including AspRS, which is involved in asparagine biosynthesis [66]. It is possible that a common function for DARS and DARS2, not strictly related to aminoacylation, could be responsible for the very similar clinical presentations in HBSL and LBSL.

Increasing evidences suggest that mt-aaRSs have a role as “chaperone,” besides their enzymatic function. According to the “channeling” hypothesis [15], interaction of mitochondrial tRNA with proteins, including mt-aaRSs, are not only necessary for tRNA synthesis, maturation, and function but also to protect tRNAs from degradation. Rapid turnover of free mt-tRNAs, not aminoacylated or bound to any protein partner, provides a general mechanism for the quality control of the tRNA pool within the human mitochondria. The data obtained from studies on mutated mt-tRNA support this hypothesis: pathogenic mutations in mt-tRNA are usually associated with a reduction in tRNA amount and a decrease of the aminoacylated form [15]. Similar indications came from studies on patients with *aaRS2 *mutations. In mutant *RARS2 *fibroblasts, the amount of tRNA^Arg^ transcript is low, but it is almost fully acylated. A residual ArgRS activity is probably present that could aminoacylate a small portion of the tRNAs^Arg^, whereas the uncharged tRNAs become unstable and are degraded [31]. In lymphocytes from patients bearing a homozygous *SARS2* mutation, the amount of tRNA^Ser(AGY)^ was strongly reduced and fully nonacylated, whereas the amount of tRNA^Ser(UCN)^ isoacceptor was nearly normal. The authors concluded that mutation in *SARS2* significantly impairs the ability of the enzyme to aminoacylate selectively the tRNA^Ser(AGY)^, leading to degradation of the uncharged tRNA molecules [36]. Therefore, mutations affecting either mt-tRNAs or mt-aaRSs, perturbing their “stabilizing” interaction, lead to decreased tRNA levels.

Additional findings in support to the channeling theory have been obtained in both yeast and human cell lines [15, 67, 68]: the overexpression of cognate, or even noncognate [69], mt-aaRSs has been demonstrated to be able to reduce/correct the deleterious effects of some mt-tRNA point mutations, leading to partial recovery of the steady-state levels of mutated tRNAs. Notably, the enzymatic activity of mt-aaRSs seems to be not required for its rescuing effect. In yeast carrying tRNA mutations, the recovery has also been obtained by overexpression of an isolated C-terminal portion of yeast LeuRS, lacking the catalytic domain [70]; it has been proposed that this polypeptide may act as a chaperone, by directly interacting with mutant mt-tRNAs, making them less prone to degradation or maintaining them in a structural conformation fostered to react with the endogenous mt-LeuRS.

## 7. Conclusions

Increasing evidences suggest that the pathophysiology of mt-aaRSs-associated disorders is driven by processes and interactions that are not yet fully understood. For instance, tissue-specific proteins that could interact differentially with the wild-type or mutant mt-aaRSs, by inhibiting the enzymatic activity or any additional function, might be identified.

In general, mt-aaRSs are relatively poorly characterized enzymes but with growing importance in human mitochondrial medicine. Generation of animal models will allow us to better understand mt-aaRSs functions, interactions, and possibly tissue specificity. These studies will surely provide important knowledge about the pathogenetic role of synthetases in the different human diseases.

## Figures and Tables

**Table 1:** Clinical and radiological phenotypes associated with different *aaRS2* mutations.

	OMIM (gene)	Protein	Clinical picture	OMIM (phenotype)	Age at onset	MRI pattern	Reported cases	References
*DARS2 *	∗610956	mt aspartyl-tRNA synthetase	Cerebellar ataxia, spasticity, dorsal column dysfunction, cognitive impairment	#611105	Childhood/adulthood	Leukoencephalopathy with brain stem and spinal cord involvement and lactate elevation (LBSL)	>30 families	[17–24]

*RARS2 *	∗611524	mt arginyl-tRNA synthetase	Encephalopathy with lethargia, hypotonia, epilepsy, and microcephaly	#611523	Perinatal	Pontocerebellar hypoplasia, brain stem thinning	>10 families	[25–28]

*YARS2 *	∗610957	mt tyrosyl-tRNA synthetase	Myopathy, lactic acidosis, and sideroblastic anemia (MLASA)	#613561	Childhood	None	3 families	[29–31]

*SARS2 *	∗612804	mt seryl-tRNA synthetase	Hyperuricemia, pulmonary hypertension, renal failure, and alkalosis (HUPRA)	#613845	Perinatal	None	4 families	[32]

*AARS2 *	∗612035	mt alanyl-tRNA synthetase	Hypertrophic cardiomyopathy, delayed motor development, cerebellar ataxia	#612035	Childhood	None	7 families	[33, 34]

*MARS2 *	∗609728	mt methionyl-tRNA synthetase	Autosomal recessive spastic ataxia	/	Childhood/adulthood	Cerebellar atrophy and white matter alterations, thin corpus callosum	1 family	[35]

*HARS2 *	∗600783	mt histidyl-tRNA synthetase	Sensorineural hearing loss and ovarian dysgenesis (Perrault syndrome)	#614926	Childhood/adulthood	None	1 family	[36]

*LARS2 *	∗604544	mt leucyl-tRNA synthetase	Sensorineural hearing loss and ovarian dysgenesis (Perrault syndrome)	#615300	Childhood/adulthood	None	1 family	[37]

*FARS2 *	∗611592	mt phenylalanine-tRNA synthetase	Epileptic encephalopathy, liver disease, and lactic acidosis	#614946	Perinatal	Cerebral and cerebellar, brain stem and basal ganglia atrophy	3 families	[38, 39]

*EARS2 *	∗612799	mt glutamyl-tRNA synthetase	Global developmental delay or arrest, epilepsy, dystonia, spasticity, and high lactate	#614924	Early childhood	Leukoencephalopathy with thalamus and brain stem involvement and high lactate (LTBL)	12 families	[40, 41]

*VARS2 *	∗612802	mt valyl-tRNA synthetase	Psychomotor delay, seizures, facial dysmorphism, lactic acidosis	/	Childhood	Hyperintense lesions in the insula and frontotemporal right cortex	1 family	[42]

*TARS2 *	∗612805	mt threonyl-tRNA synthetase	Psychomotor delay, hypotonia	/	Perinatal/early childhood	Thin corpus callosum, bilateral lesion of the pallidum	1 family	[42]

*GARS *	∗600287	Glycyl-tRNA synthetase	Charcot-Marie-Tooth (CMT) disease 2D or distal hereditary motor neuropathy VA	#601472, #600794	Childhood/adulthood	None	>8 families	[43–50]

*KARS *	∗601421	Lysyl-tRNA synthetase	Autosomal recessive CMT (intermediate, B)	#613641	Childhood/adulthood	None	4 families	[51, 52]
